# Gram-Negative Bacteria *Salmonella typhimurium* Boost Leukotriene Synthesis Induced by Chemoattractant fMLP to Stimulate Neutrophil Swarming

**DOI:** 10.3389/fphar.2021.814113

**Published:** 2022-01-04

**Authors:** Ekaterina A. Golenkina, Svetlana I. Galkina, Olga Pletjushkina, Boris Chernyak, Tatjana V. Gaponova, Yulia M. Romanova, Galina F. Sud’ina

**Affiliations:** ^1^ Belozersky Institute of Physico-Chemical Biology, Lomonosov Moscow State University, Moscow, Russia; ^2^ National Research Center for Hematology, Russia Federation Ministry of Public Health, Moscow, Russia; ^3^ Gamaleya National Research Centre of Epidemiology and Microbiology, Moscow, Russia

**Keywords:** neutrophil, bacteria *Salmonella typhimurium*, intracellular calcium, 5-lipoxygenase, leukotriene B4, neutrophil swarming

## Abstract

Leukotriene synthesis in neutrophils is critical for host survival during infection. In particular, leukotriene B_4_ (LTB_4_) is a powerful neutrophil chemoattractant that plays a crucial role in neutrophil swarming. In this work, we demonstrated that preincubation of human neutrophils with *Salmonella typhimurium* strongly stimulated LTB_4_ production induced by the bacterial chemoattractant, peptide N-formyl-L-methionyl-L-leucyl-l-phenylalanine (fMLP), while the reverse sequence of additions was ineffective. Preincubation with bacterial lipopolysaccharide or yeast polysaccharide zymosan particles gives weaker effect on fMLP-induced LTB_4_ production. Activation of 5-lipoxygenase (5-LOX), a key enzyme in leukotrienes biosynthesis, depends on rise of cytosolic concentration of Ca^2+^ and on translocation of the enzyme to the nuclear membrane. Both processes were stimulated by *S. typhimurium*. With an increase in the bacteria:neutrophil ratio, the transformation of LTB_4_ to ω-OH-LTB_4_ was suppressed, which further supported increased concentration of LTB_4_. These data indicate that in neutrophils gathered around bacterial clusters, LTB_4_ production is stimulated and at the same time its transformation is suppressed, which promotes neutrophil swarming and elimination of pathogens simultaneously.

## Introduction

Neutrophils (polymorphonuclear leukocytes, PMNLs) are the most abundant leukocytes circulating in mammalian blood. They are the first immune cells recruited by invading pathogens or damaged cells, playing a central role in both inflammation and host defense (Metschnikoff 1891; Kobayashi et al., 2018). Peptides containing N-formylated methionine, which is a hallmark of bacterial translation, are the primary neutrophil chemoattractants during bacterial infection (Snyderman and Pike 1984). Neutrophils express formyl peptide receptors (FPR1 and FPR2) for these peptides (Ye et al., 2009; Dahlgren et al., 2016). N-formyl-L-methionyl-L-leucyl-l-phenylalanine (fMLP), a prototype N-formylated peptide, is a potent ligand for FPR1, a strong neutrophil chemoattractant, and macrophage activator (Schiffmann et al., 1975; Snyderman and Pike 1984; Ye et al., 2009; Dorward et al., 2015; Dahlgren et al., 2016). FPRs play a critical role in defense against bacteria by recruiting inflammatory cells to sites of infection.

Activated neutrophils penetrate the endothelium of blood vessels and infiltrate tissues to form inflammation focuses. The tissues in these focuses, as well as resident macrophages and neutrophils, themselves, release secondary chemoattractants to attract more leukocytes and amplify inflammation. The first secondary chemoattractant produced in inflammation focuses is leukotriene B_4_ (LTB_4_) (Brandt and Serezani 2017), and neutrophils from mice lacking the specific LTB_4_ receptor BLT1 were not able to swarm and cluster to a focal damage site (Lammermann et al., 2013). The synthesis of LTB_4_ from arachidonic acid is catalyzed by 5-lipoxygenase (5-LOX), which is activated by various inflammatory mediators (Radmark and Samuelsson 2010; Radmark et al., 2015; Haeggstrom 2018).

Neutrophil production of LTB_4_ and the release of another chemoattractant, chemokine CXCL2 (C-X-C motif chemokine ligand 2), is responsible for the collective coordinated behavior of neutrophils, called swarming, which is important for protection against severe pathogen infection (Lammermann et al., 2013; de Oliveira et al., 2016; Rocha-Gregg and Huttenlocher 2021). During swarming hundreds of individual neutrophils respond with coordinated chemotaxis and self-amplified clusters formation. Early recruitment of neutrophil is initiated by pathogen-associated molecular patterns (PAMPs), including N-formyl peptides, and damage-associated molecular patterns (DAMPs), which are released mainly from damaged cells (Venereau et al., 2015). Some of the early recruited (“pioneer”) neutrophils are activated to produce LTB_4_ (Lammermann et al., 2013). LTB_4_, in turn, dramatically amplify fMLP-induced neutrophil polarization and chemotaxis (Afonso et al., 2012), completing the self-amplification cycle. In addition, LTB_4_ stimulates bacterial phagocytosis by neutrophils (Mancuso et al., 2001).

Neutrophil swarming provides a significant boost to the accumulation of neutrophils at sites of injury or infection and serves for engulfing microbes and their clusters that are too large for individual neutrophils to kill (Hopke et al., 2020). Swarming is only triggered against targets above a certain size threshold (Reategui et al., 2017). Swarming of neutrophils can exacerbate inflammation and tissue damage, so a mechanism is needed to control the excessive swarming. Very recently, it was found that desensitization of G protein-coupled receptors (including FPRs) significantly contributes to the self-limitation of swarming (Kienle et al., 2021). Another possible control mechanism was described much earlier, when it was shown that fMLP is degraded at the cell surface of neutrophils (Yuli and Snyderman 1986).

Understanding the mechanisms that control the formation of LTB_4_, an important stimulus for swarming, when exposed to the chemoattractant N-formyl peptides in the presence of bacteria, will provide insight into the prevention and treatment of inflammatory diseases. In this study, we analyzed fMLP-induced leukotriene synthesis modulated by the interaction of neutrophils with the Gram-negative bacteria *Salmonella typhimurium*.

## Materials and Methods

Hank’s balanced salt solution with calcium and magnesium but without Phenol Red and sodium hydrogen carbonate (HBSS), Dulbecco’s phosphate-buffered saline (PBS) with magnesium but without calcium, fibrinogen from human plasma, N-Formyl-L-methionyl-L-Leucyl-l-Phenylalanine (fMLP) and N-t-Boc-L-Methionyl-L-Leucyl-l-Phenylalanine (Boc-MLP) were purchased from Sigma (Steinheim, Germany). Dextran T-500 was from Pharmacosmos (Holbæk, Denmark).

### Neutrophil Isolation

Human polymorphonuclear leukocytes (PMNLs) were isolated from freshly drawn blood with citrate anticoagulant. Experimental and the subject consent procedures were approved by the Bioethics Committee of the Lomonosov Moscow State University, Application # 6-h, version 3, Bioethics Commission meeting # 131-d held on May 31, 2021. Leukocyte-rich plasma was prepared from the donor blood by sedimentation in the presence of T-500 Dextran. Granulocytes were obtained as described (Aleksandrov et al., 2006). Cell viability was checked by trypan blue exclusion. PMNLs (96–97% purity, 98–99% viability) were stored at room temperature in Dulbecco’s PBS containing 1 mg/ml glucose (no CaCl_2_).

### Preparation of Bacteria

Bacteria (*S. typhimurium* IE 147 strain) were obtained from the Collection of Gamaleya National Research Center of Epidemiology and Microbiology (Moscow, Russia). Bacteria were grown in Luria–Bertani broth to a concentration of 1 × 10^9^ colony-forming units (CFU)/mL. In this study not opsonized and opsonized bacteria were used. Bacteria were opsonized immediately before the experiment for 30 min in 20% (v/v) fresh serum from the same donor whose blood was used to isolate neutrophils. Repeated centrifugation in Dulbecco’s solution was used to wash the bacteria.

### Determination of 5-LOX Product Formation in Cells

PMNLs (1 × 10^7^/6 ml HBSS/Hepes) were preincubated at 37°C in CO_2_ incubator for 10 min, then bacteria, or zymosan, or reagents were added, as indicated. The incubation was stopped by adding of an equal volume of methanol (−18°C) with 90 ng prostaglandin B_2_ as internal standard. The water-methanol extracts stored at −18°C. After centrifugation, the water-methanol extracts were purified by solid-phase extraction on Sep-Pak C18 cartridges (500 mg; Macherey-Nagel, Dueren, Germany), as described (Viryasova et al., 2016). The purified samples were injected into a 5 μm, 250 × 4.6 mm Nucleosil^®^ C18 column (Macherey-Nagel GmbH) and subjected to RP HPLC. Products of the 5-LOX pathway included 5S, 12R-dihydroxy-6,14-*cis*-8,10-*trans*-eicosatetraenoic acid (LTB_4_), iso-LTB_4_ (5S, 12SR-all-*trans*-diHETE) (t-LTB_4_), ω-OH-LTB_4_, ω-COOH-LTB_4_ and 5S-hydroxy-6-*trans*-8,11,14-*cis*-eicosatetraenoic acid (5-HETE). Major 5-LOX metabolites were identified by comparing retention times with those of known compounds, as previously described (Viryasova et al., 2014). The compounds were quantified by comparison of peak areas with the internal standard prostaglandin B2 (Cayman Chemical, Ann Arbor, United States).

### Analysis of 5-LOX Subcellular Localization by Immunofluorescence Microscopy

PMNLs (2 × 10^6^/mL HBSS/HEPES) were incubated without stimuli, as well as in the presence of non-opsonized bacteria, fMLP, or under conditions of sequential addition of bacteria and the formyl peptide. The incubation time was 20 min, additional stimulation with fMLP took another 5 min. The treatment was carried out at 37°С in microcentrifuge tubes with continuous stirring. After the expiration of incubation time, treated suspensions were placed on uncoated glasses of confocal dishes for 5 min, the supernatants were carefully removed, and the settled cells were fixed with 4% paraformaldehyde solution for 10 min at room temperature. Fixed cells were permeabilized with 0.1% Triton X-100 for 10 min at room temperature, followed by blocking with 1% BSA in PBS. The samples were then incubated overnight at 4 °C with rabbit polyclonal anti-5-LOX antibody (1:50 in blocking solution) (Cayman Chemical, Michigan, United States). Samples were rinsed with blocking solution, followed by staining with Oregon Green 488 goat anti-rabbit antibodies (1:100 in blocking solution) (Thermo Fisher Scientific, Waltham, MA, United States) for 1 h at 4°C. DNA was stained with 0.5 μg/ml Hoechst 33342 (Thermo Fisher Scientific, Waltham, MA, United States). The cells were visualized by a Zeiss Axiovert 200M fluorescence microscope equipped with 100× oil immersion objective.

### Calcium Influx Analysis

Freshly isolated PMNLs were loaded with Fluo-3, AM dye (Thermo Fisher Scientific, Waltham, MA United States) accordingly to manufacturer’s protocol. Briefly, cells were incubated with 5 μM Fluo-3 AM ester in Ca^2+^-free Dulbecco′s PBS for 60 min at room temperature, followed by washing with PBS. The labeled cells were then seeded in fibrinogen-coated 96-well plates (1 × 10^6^/ml of HBSS/HEPES) and incubated according to the experimental protocol at 37°C in 5% CO_2_. A suspension of unstained cells was used as blank. Changes in fluorescence intensity upon excitation at 488 nm and emission at 535 were monitored for at least 70 s after each stimulus injection. Manipulations were performed on a ClarioStar fluorescence microplate reader (BMG Labtech, Cary, NC, United States).

### Scanning Electron Microscopy

For scanning electron microscopy, cells were fixed for 30 min in 2.5% glutaraldehyde, postfixed for 15 min with 1% osmium tetroxide in 0.1 M cacodylate (pH 7.3), dehydrated in an acetone series, and processed by conventional scanning electron microscopic techniques, as described (Galkina et al., 2015).

### Statistics

Results are presented as mean ± SEM. Analysis of statistical significance for multiple comparisons was performed using GraphPad Prism 9.2.0 software. Differences with *p*-values <0.05 were considered statistically significant.

## Results

### fMLP Boosts Leukotriene Synthesis in PMNL Pre-exposed to Bacteria

**FIGURE 1:**
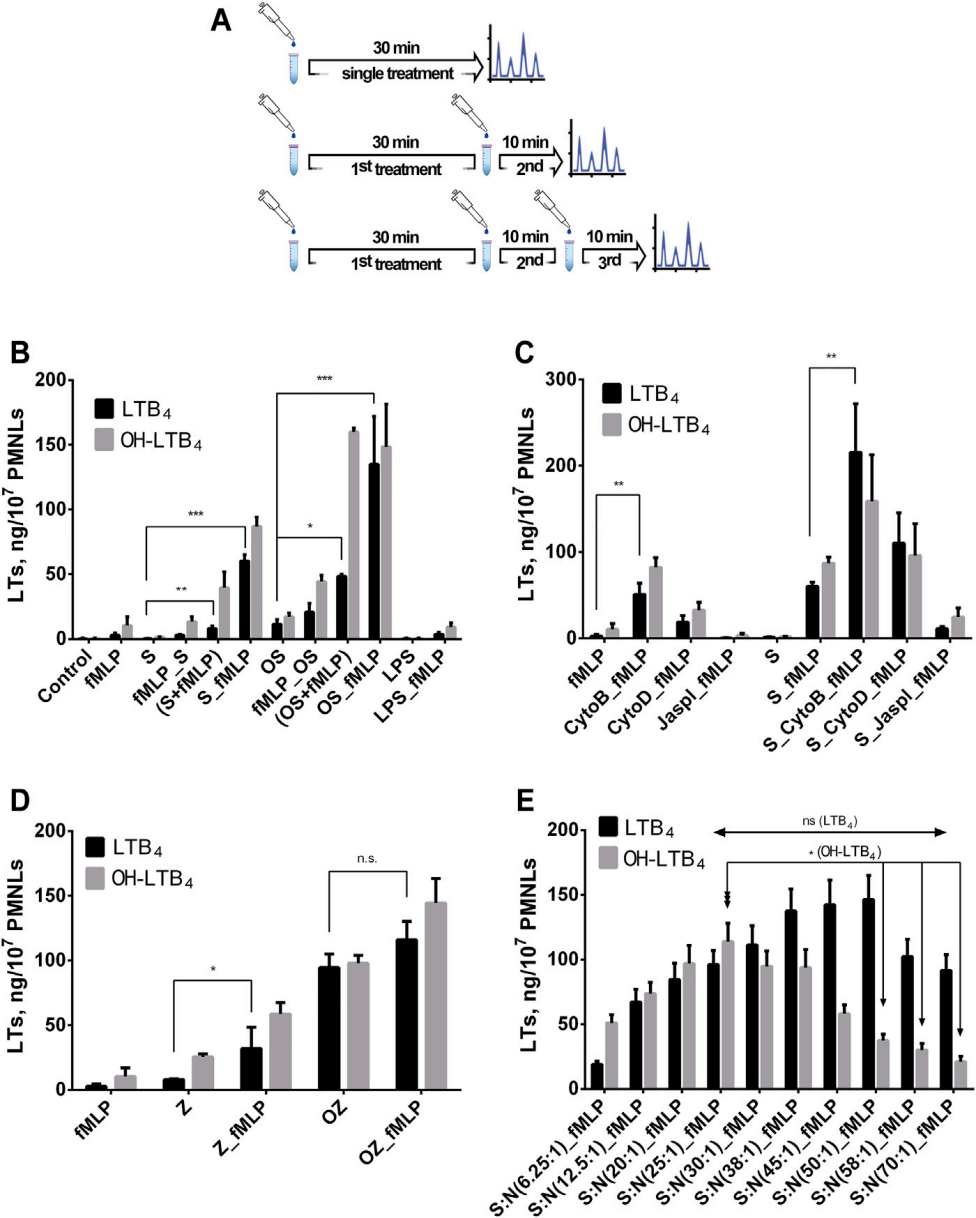
Effect of *Salmonella typhimurium* or zymosan on fMLP-induced leukotriene synthesis in human neutrophils. Before treatment, PMNLs (0.9–1.0) x10^7^/6 ml were pre-incubated for 10 min at 37°C, 5% CO_2_. **(A)** Timing options for treatments are presented. **(B,C)**—(the ratio of bacteria:PMNLs ∼25:1). **(B)** At single treatment, control (no additives), or fMLP (0.1 µM), or S/OS, or bacteria plus fMLP (S/OS + fMLP), or LPS (2 μg/ml) were added for 30 min. At complex treatment, bacteria, or fMLP, or LPS were added as first stimulus and S, OS or fMLP as the second. Hereinafter, on the *X*-axis, sequential stimuli are labeled, listed in the order of addition and separated by an underscore. Values present mean ± SEM of five independent experiments performed in duplicate. **(D)** At single treatment, fMLP (0.1 µM), or Z/OZ (0.4 mg/ml) were added for 30 min. At complex treatment, Z/OZ (0.4 mg/ml) were added as the first stimulus, and then fMLP (0.1 µM) as the second, as indicated. Values indicate mean ± SEM of three independent experiments performed in duplicate. **(C)** At single treatment, fMLP (0.1 µM), or S were added for 30min. At complex treatment, no additives (left panel) or bacteria (right panel) were added for 30 min, then a second stimulation was performed with Cyto B (5 µM) or Cyto D (10 µM) or Jaspl (0.5 µM) followed by the third treatment with fMLP (0.1 µM) as indicated. Values indicate mean ± SEM of five independent experiments performed in duplicate. **(E)** Leukotriene synthesis in human neutrophils exposed to *Salmonella typhimurium* (first treatment) followed by fMLP (0.1 µM) addition for 10 min, at various bacterial load. The ratio of bacteria (S):PMNLs (N) is indicated. Values present mean ± SEM of five independent experiments performed in duplicate. The 5-LOX products were analyzed using HPLC, and data for LTB_4_ and ω-OH-LTB_4_ are presented. *p < 0.05, **p < 0.01, ***p < 0.001 for pairs of data compared as indicated by two-way ANOVA followed by Tukey’s multiple comparison test.

We observed a strong stimulation of leukotriene synthesis induced by formyl-peptide when neutrophils were pre-activated by either opsonized (OS) or non-opsonized *S. typhimurium* (S). The most effective was 30 min pre-treatment with bacteria followed by 10 min with fMLP (S_fMLP and OS_fMLP; two treatments divided by lower dash) (Figure 1B). In the case of non-opsonized bacteria, the stimulating effect of fMLP was even more pronounced,—addition of fMLP to infected cells increased leukotriene synthesis by more than two orders of magnitude. With OS we had 10-fold stimulation of LT biosynthesis at fMLP adding after bacteria. The time of interaction with bacteria prior to fMLP stimulation was important; in our assay, the synthesis of leukotrienes reached its maximal level after 30–40 min of neutrophil incubation with bacteria (Supplementary Figure 1). On Supplementary Figure 1 presented all 5-LOX metabolites that we detected in our assay. The main 5-LOX products are LTB_4_ and ω-OH-LTB_4._

Pre-exposure to LPS, the surface marker of bacteria, was not efficient (Figure 1B). Importantly, pretreatment with fMLP did not stimulate leukotriene synthesis initiated by non-opsonized (fMLP_S) or opsonized (fMLP_OS) *S. typhimurium* (Figure 1B). When using opsonized or non-opsonized zymosan (OZ or Z) for cell pretreatment, we observed a 5-fold stimulating effect of fMLP with Z, with OZ the peptide had a less pronounced stimulating effect (Figure 1D).

It is well known that pre-treatment with Cytochalasin B (Cyto B) sharply increased 5-LOX product formation in neutrophils at fMLP exposure (Foldes-Filep and Filep 1992). However, CytoB is also known to inhibit glucose transport across the plasma membrane (Bloch 1973). Its analogue Cytochalasin D (Cyto D) also inhibits actin cytoskeleton but does not affect glucose transport (Atlas et al., 1980). This is why we used both cytochalasin’s to study the role of actin cytoskeleton in 5-LOX activation. In our experiments depolymerization of actin predisposed to greater response to fMLP (Figure 1C). Actin polymerizing agent Jasplakinolide (Jaspl) suppressed activation of leukotriene synthesis by bacteria/fMLP. fMLP receptor antagonist BocMLP inhibited 5-LOX product formation in concentration–dependent manner (Supplementary Figure 2).

The most powerful stimulus for 5-LOX activation is calcium ionophore A23187. Ionophore stimulation produces twice the amount of leukotrienes compared to the combination of bacteria and formyl peptide (Supplementary Figure 3). Pre-treatment with bacteria did not further increase the effect of A23187; fMLP added after A23187 just contributes to LTB_4_ transforming to ω-OH-LTB_4_ (Supplementary Figure 3).

Importantly, when neutrophils are exposed to bacteria followed by fMLP the profile of 5-LOX products depends on the ratio bacteria:neutrophil, and as the bacterial load increases the ω-OH-LTB_4_ and LTB_4_ ratio changes in favor of LTB_4_ (Figure 1E). These data show that high bacterial load not only increases the synthesis of LTB_4_, but also suppresses its transformation. The drop of ω-OH-LTB_4_/LTB_4_ ratio is not due to enhanced conversion of 20-OH-LTB_4_ to 20-COOH-LTB_4_, with increasing bacterial load the synthesis of 20-COOH-LTB_4_ decreased (Supplementary Figure 4).

### Bacteria Stimulate fMLP-Induced Cell Signaling, Resulting in 5-LOX Activation

**FIGURE 2:**
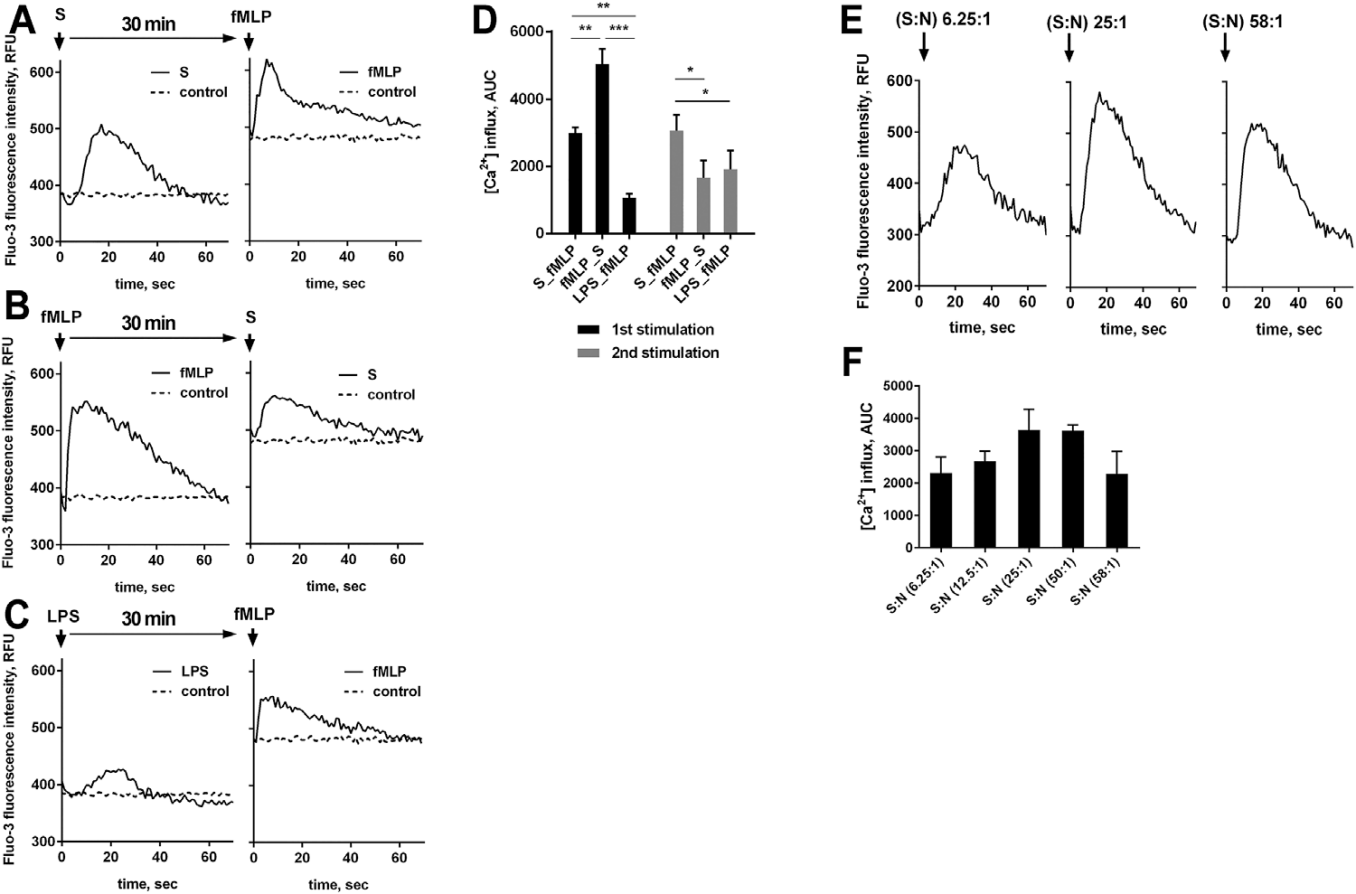
A-D. fMLP-induced increase in [Ca2+]i in the presence of bacteria or LPS. Fluo-3 AM loaded PMNLs (4 × 10^5^/sample) were cultured in fibrinogen-coated flat bottom 96-well plates in HBSS/HEPES medium at 37°C, 5% CO_2_. Cells were sequential exposed to nonopsonized *S. typhimurium* (S) (bacteria per cell ratio 25:1), LPS (2 μg/ml) and 0.1 µM fMLP with an interval of 30 min (the order of adding stimuli is shown in the figure). HBSS/HEPES was added to control samples (dotted lines on A-C). Flash kinetic of Fluo-3 fluorescence (ex. 488 nm, em. 535 nm) was monitored with 1 s interval. **(A, B, C)** Changes in [Ca^2+^]_i_ are presented as typical blank corrected Fluo-3 fluorescence kinetic curves for each type of stimulation. Diagram **(D)** show areas under curves (AUC) with control values as baseline for fluorescence obtained over 70 s after the addition of stimuli. Values indicate mean ± SEM, n = 3, *p < 0.05, **p < 0.01, ***p < 0.001 for pairs of data compared as indicated by two-way ANOVA followed by Tukey’s multiple comparison test. **(E, F)**. [Ca^2+^]_i_ changes in neutrophils following *S. typhimurium* exposure at various bacterial load. Fluo-3 AM loaded PMNLs (4 × 10^5^/sample) were cultured in fibrinogen-coated flat bottom 96-well plates in HBSS/HEPES medium at 37°C, 5% CO_2_. PMNLs were stimulated with nonopsonized bacteria with an increase in bacterial load from 6.25 to 58 bacteria per cell (as indicated). Flash kinetic of Fluo-3 fluorescence (ex. 488 nm, em. 535 nm) was monitored with 1 s interval. **(E)** Changes in [Ca^2+^]_i_ are presented as typical blank corrected Fluo-3 fluorescence kinetic curves for the average (25: 1) and extreme values of the studied range of bacterial load. AUC’s for fluorescence obtained over 70 s after the addition of bacteria are represented on **(F)** (values indicate mean ± SEM, n = 3).

Efficient assembly and functioning of the enzymatic apparatus for the synthesis of leukotrienes requires an increase in the concentration of free Ca^2+^ in the cytoplasm ([Ca^2+^]_i_). We investigated changes in [Ca^2+^]_i_ in response to the studied stimuli and their combinations. It was shown that the sequential stimulation by bacteria and fMLP optimal for induction of LTB_4_ (Figure 1B) was accompanied with maximal rise in [Ca^2+^] at second treatment (Figure 2A). Rise in the [Ca^2+^]_i_ in response to formyl-peptide decreased when neutrophils were primed with bacterial LPS (Figure 2C). In non-primed cells, fMLP produced maximal [Ca^2+^]_i_ jump (Figures 2B,D).

When evaluating the effect of bacteria on changes in [Ca^2+^]_i_, we found that as the ratio bacteria:neutrophils increased, the amplitude of calcium pulses increased, with a subsequent decrease (Figures 2E,F). The bacterial load threshold beyond which calcium release is suppressed coincides with the results of the analysis of leukotriene synthesis, according to which exceeding the 50:1 ratio promotes the predominant accumulation of LTB_4_, with decreasing the sum of leukotrienes (Figure 1E).

**FIGURE 3:**
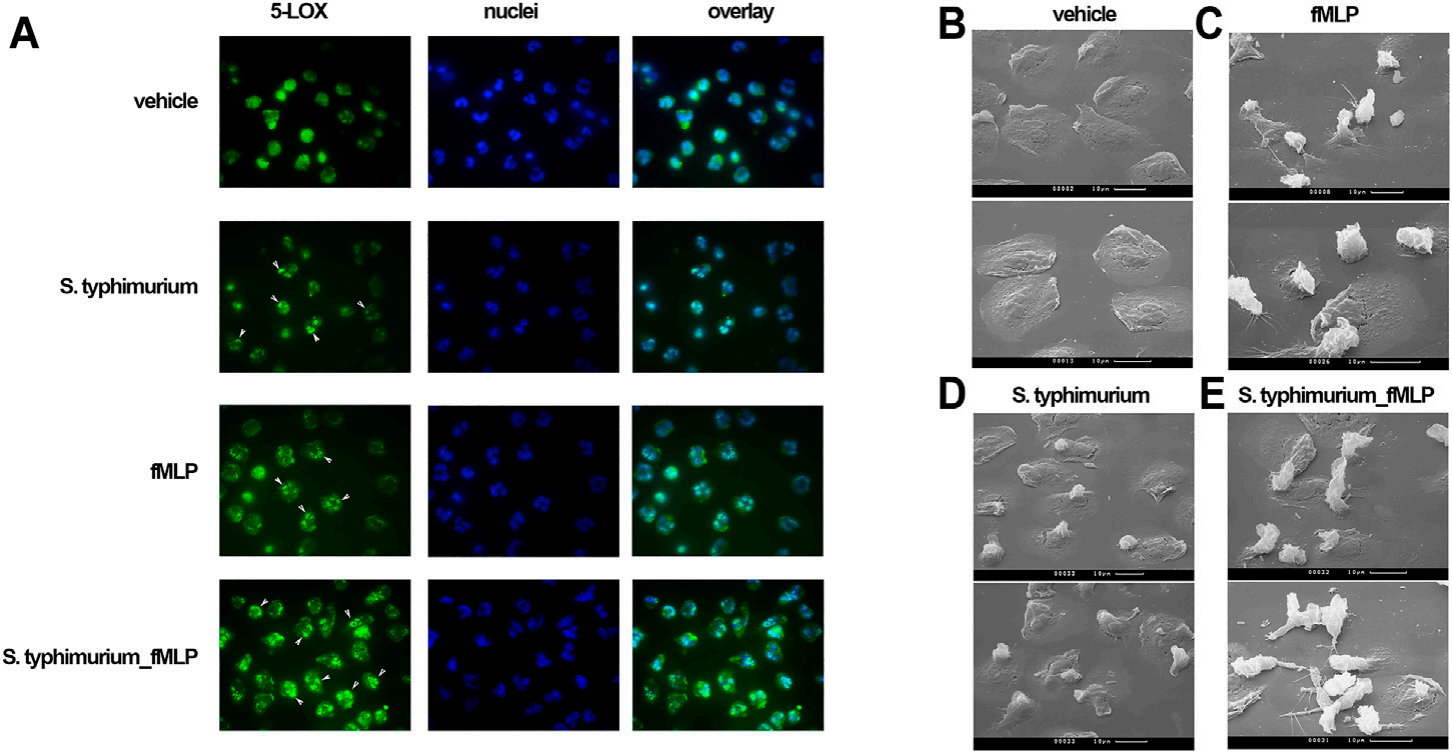
**(A)** Subcellular localization of 5-LOX by immunostaining. PMNLs (2 × 10^6^/mL HBSS/HEPES) were incubated without additived (vehicle), with nonopsonized *S. typhimurium* (25:1 bacteria per cell ratio), with 0.1 µM fMLP or under sequential stimulation with these agents at intervals of 20 min. The final incubation with fMLP in this case lasted 5 min. At the end of the incubation, the cells were fixed and stained for 5-LOX (green). Samples were also stained for DNA with Hoechst (blue). **(B–E)**. Scanning electron microscopy appearance of neutrophils in co-incubation with bacteria and fMLP. PMNLs (10^6^/ml) in HBSS/HEPES medium were plated on coverslips and incubated for 20 min at 37°C in the medium **(B, C)**, or with nonopsonized *S. typhimurium* (25:1 bacteria per cell ratio) **(D, E)**, followed by adding fMLP (0.1 µM) for 5 min **(C, E)**.

Translocation of 5-LOX to the nuclear membrane is required for 5-LOX activity (Luo et al., 2003), and is initiated by an increase in [Ca^2+^]_i_ (Kulkarni et al., 2002). Co-localization of lipoxygenase with 5-LOX activating protein (FLAP) on the nuclear membrane appears to be a very effective mechanism for the rapid regulation of leukotriene synthesis (Newcomer and Gilbert 2010). 5-LOX translocation was assessed by immunofluorescence microscopy. In non-activated control cells (Figure 3, vehicle), 5-LOX is uniformly distributed over the cytoplasm. Short-term (30 min) incubation of cells with either bacteria or formyl-peptide leads to the appearance of 5-LOX clusters in perinuclear area of some cells (indicated with white arrows). Sequential stimulation with bacteria and fMLP resulted in 5-LOX translocation in almost all cells in the sample (bottom row, white arrows). The 5-LOX translocation may be mediated by the effect of bacteria on mitogen-activated protein kinases (MAPK). In particular, we observed strong inhibition of LT synthesis by the ERK kinase inhibitor U0126 (Supplementary Figure 5).

The study of cellular morphology showed appearance of intercellular contacts in the presence of bacteria and fMLP (Figure 3E). Recently, it was found that clustering of neutrophils during swarming allows the propagation of Ca^2+^ signals via connexin-43 hemichannels (Poplimont et al., 2020). These channels were formed in gap junctions. LTB_4_ in our model leads to the formation of cell contacts, which may influence Ca^2+^ signaling in cells and possible propagating of Ca^2+^ signals in dense swarming.

## Discussion

Several pathogens have been shown to activate 5-LOX, and the resulting synthesis of leukotrienes is critical for host survival (Yamamoto et al., 1993; Caffrey-Carr et al., 2017; Werz et al., 2018). In the infection-on-a-chip model, it has been shown that the environmental fungal pathogen *Aspergillus fumigatus* induces LTB_4_ secretion by neutrophils (Hind et al., 2021). More recently, it was reported that the common opportunistic fungal pathogen *Candida albicans* induced 5-LOX activation and LTB_4_ formation in neutrophils when hyphae are formed (Fischer et al., 2021). In this model [Ca^2+^]_i_ mobilization and p38 MAPK activation followed by 5-LOX translocation to the nuclear membrane were observed.

Gram-negative bacteria *Escherichia coli* and Gram-positive bacteria *Staphylococcus aureus* stimulated 5-LOX in M1 macrophages (Werz et al., 2018). Pathogenic *S. aureus*, but not exotoxin-deficient strains, induced 5-LOX activation in HEK293 cells stably transfected with human 5-LOX and FLAP (HEK_5- LOX/FLAP) (Romp et al., 2020). Interestingly, one of the *S. aureus* exotoxins, amphipathic α-helical phenol-soluble modulin (PSM), stimulated 5-LOX in human neutrophils. This effect was prevented by a selective antagonist of FPR2 receptor, indicating that this receptor, which recognizes not only N-formyl peptides, but also the arachidonic acid metabolite lipoxin A4 (Dahlgren et al., 2016), mediates leukotriene biosynthesis. Our earlier study demonstrated that *S. typhimurium* induced insignificant LTB_4_ production, while opsonized bacteria stimulated LTB_4_ production to a level of 5–20 ng/10^7^ PMNLs (Golenkina et al., 2011). fMLP was unable to activate 5-LOX in neutrophils until the cells were pretreated with CytB (Foldes-Filep and Filep 1992). These results were confirmed in the present study (Figure 1 B, C). Interestingly, it was shown that CytD, which is a more specific inhibitor of actin cytoskeleton than CytB, also stimulated synthesis of leukotrienes although the effect was less pronounced (Figure 1C). Moreover, we demonstrated for the first time that preincubation of human neutrophils with *S. typhimurium* strongly stimulated fMLP-induced leukotriene production. The reverse sequence of additions was found to be ineffective (Figure 1B). Treatment of neutrophils with not opsonized zymosan slightly stimulated fMLP-induced leukotriene synthesis (Figure 1D). LPS did not result in enhanced leukotriene production in response to fMLP, as published (Doerfler et al., 1989). LPS can prime for enhanced production of leukotrienes in fMLP-stimulated neutrophils in the presence of serum (Surette et al., 1993; Brideau et al., 1999).

Exposure of neutrophils to various pro-inflammatory stimuli causes synergistic functional responses to fMLP, a phenomenon known as priming (Miralda et al., 2017). It has been shown that the production of leukotrienes in neutrophils is the subject of the priming by proinflammatory cytokines. Both granulocyte-macrophage colony-stimulating factor (GM-CSF) (DiPersio et al., 1988) or tumor necrosis factor (TNF) (Bauldry et al., 1991), which by themselves do not induce LTB_4_ formation, strongly stimulate fMLP-induced LTB_4_ production. Moreover, it was reported that GM-CSF primed neutrophils to LTB_4_ production induced by A23187 (DiPersio et al., 1988). The mechanisms of cytokine priming of leukotriene synthesis have not been elucidated. It was shown that TNF has no direct effect on either the activation of phospholipase A2 and arachidonic acid mobilization, or on [Ca^2+^]_i_ basal, or on increased by fMLP (Bauldry et al., 1991). Later, it was demonstrated that GM-CSF and TNF have a very strong priming effect on the synthesis of leukotrienes in whole blood, stimulated by fMLP (Palmantier et al., 1994). The effects of the two cytokines on LTB_4_ synthesis in whole blood were additive, indicating different priming mechanisms.

Some bacteria caused marked priming of fMLP-induced production of reactive oxygen species (ROS) catalyzed by NADPH oxidase in neutrophils. For example, early studies have shown that protease-sensitive components of ultrasonicated *Helicobacter pylori* with an apparent molecular weight of 25–35 kDa (Nielsen and Andersen 1992) and lipopolysaccharide (LPS) of the cell wall of *E. coli* (Karlsson et al., 1995) stimulate fMLP-induced burst of extracellular chemiluminescence, reporting ROS production. Infection of human neutrophils with intracellular Gram-negative bacteria *Anaplasma phagocytophilum* or the eucaryotic parasite *Leishmania major* leads to a significantly more active formation of LTB_4_, induced by combined action of fMLP and LPS, than in uninfected neutrophils (Plagge and Laskay 2017).

To our knowledge, the activation of fMLP-induced leukotriene synthesis in neutrophils by extracellular bacteria was first described in this study. We observed that *S. typhimurium* (opsonized or not) stimulated the synthesis of leukotrienes at least 10-folds (Figure 1B). Activation of 5-LOX correlates with its translocation to the nucleus (Figure 3). Since the location of FLAP at the nuclear membrane of neutrophils has been proven in many previous reports (Brock et al., 1994; Mandal et al., 2008; Bair et al., 2012; Gerstmeier et al., 2016; Fischer et al., 2021), the effect of bacteria and fMLP on 5-LOX translocation provides co-localization of 5-LOX and FLAP, which is critical for 5-LOX activity.

5-LOX activation is calcium dependent (Kulkarni et al., 2002). The increase in [Ca^2+^]_i_ caused by fMLP was more pronounced without pretreatment with bacteria (Figures 2B,D). And though the total Ca^2+^ influx was practically independent of the sequence of addition of bacteria and fMLP (Figure 2D), in the second treatment, calcium response to fMLP is higher in cells pre-exposed to bacteria (Figure 2). It can be assumed that bacteria, probably, protect formyl peptide receptors (FPRs) from desensitization, which is regulated by cytoskeleton (Jesaitis and Klotz 1993; Klotz and Jesaitis 1994). It was shown that shortly after binding of fMLP to its neutrophil receptor, the ligand-receptor complex becomes associated with the cytoskeleton (Jesaitis et al., 1993; Klotz et al., 1994), and cytochalasin B prevents desensitization (Harbecke et al., 1997). It should be noticed that bacteria and cytochalasins B and D synergistically enhanced LTB_4_ synthesis (Figure 1C), which indicates the involvement of multiple signaling pathways in priming mechanisms.

We observed the formation of cell-cell contacts by neutrophils sequentially stimulated by bacteria and fMLP (Figure 3E). Earlier it was shown the role of LTB_4_ in the aggregation of human neutrophils induced by the chemotactic peptide fMLP (Beckman et al., 1985). We suggest that increased LTB_4_ production results in formation of loose neutrophil clusters, where local concentrations of LTB_4_ and the other mediators increase to stimulate further clustering and swarming. It was recently found that neutrophils form gap junctions during swarming, which make possible the propagation of Ca^2+^ signals through the connexin-43 hemichannels (Poplimont et al., 2020). Intercellular exchange of Ca^2+^ signals, along with LTB_4_ and other signaling molecules, is critical for dense swarms’ formation and fighting with pathogens.

In severe inflammation, aging neutrophils evading uptake by macrophages produced an increased amount of chemoattractants 5-oxo-ETE and LTB_4_, which leads to a delayed resolution or exacerbation of the inflammatory process;—they have reduced LTB_4_ 20-hydroxylase (ω-OH-) activity (Graham et al., 2009). Neutrophil omega-hydroxylase converts LTB_4_ to 20-hydroxy (ω-OH) LTB_4_ (Powell 1984), which is a less potent chemoattractant than LTB_4_. It is known that bacterial uptake modulates the inflammatory responses of granulocytes. Bacteria-pretreated PMNLs, after further stimulation with zymosan, had decreased transformation of LTB_4_ to ω-OH-LTB_4_ (Grone et al., 1992). PMNLs after phagocytosis of bacteria showed a partially or completely suppressed respiratory burst (Grone et al., 1992). 20-OH- and 20-COOH-LTB_4_ bind to the BLT1 receptor with high affinity but activate neutrophils to a much lower extent than LTB_4_ (Archambault et al., 2019), so ω-OH-LTB_4_ and ω-COOH-LTB_4_ act as natural inhibitors of LTB_4_-mediated responses. Thus, preventing LTB_4_ ω-oxidation might result in increased innate immunity and granulocyte functions. Studies with subpopulations of human blood cells and human plasma clearly indicated that the polymorphonuclear leukocytes were the main source of enzymic activity for the omega-oxidation of LTB_4_ (Nadeau et al., 1984).

**SCHEME 1:**
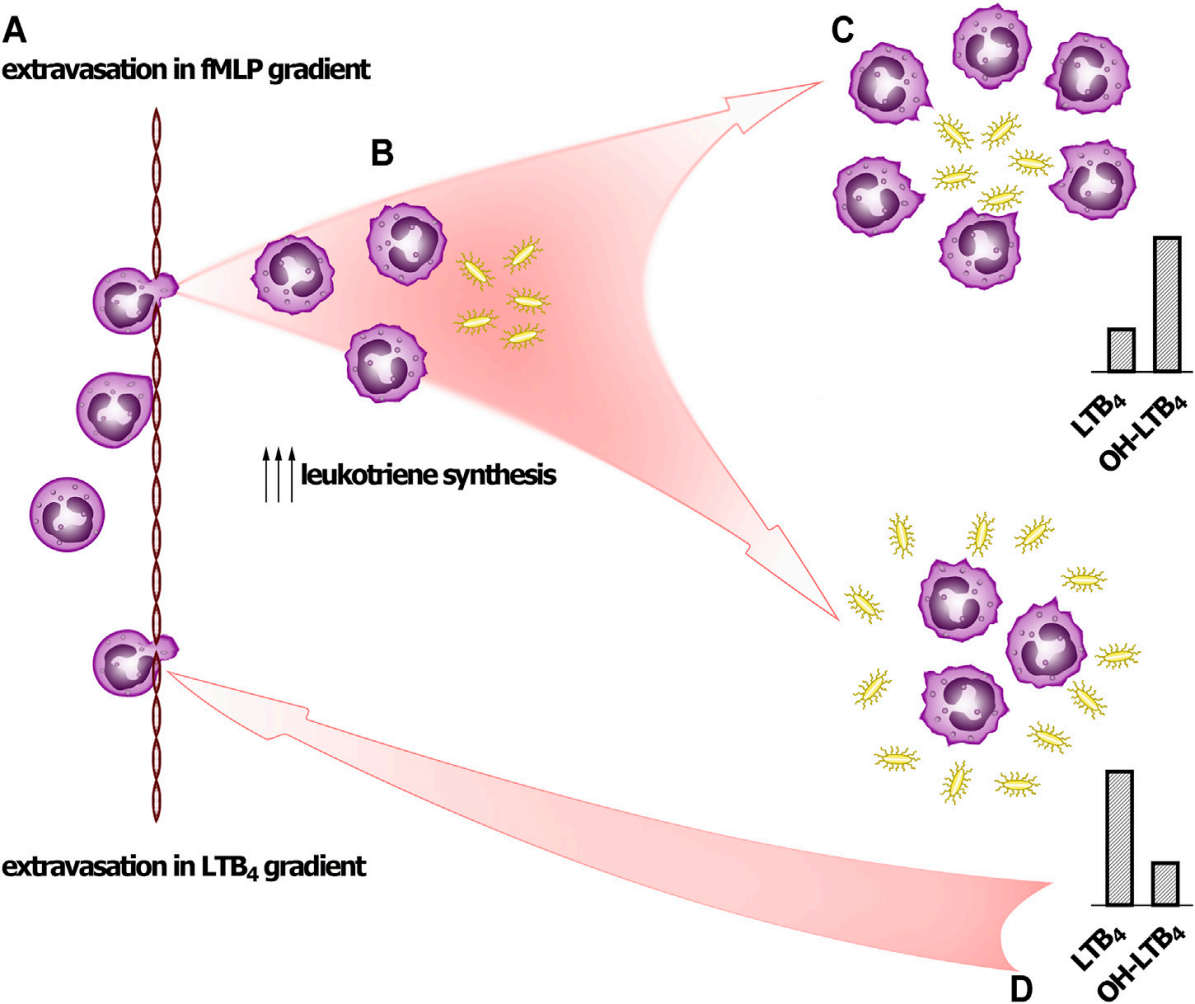
Signal self-amplification to stimulate neutrophil swarming. **(A)** Pioneer PMNLs meet fMLP and move to bacteria. **(B)** PMNLs meet bacteria and pathogen-associated molecular patterns (PAMPs), including formyl-methionyl peptides. The leukotriene synthesis is accelerated, and neutrophils swarm at site of infection. **(C)** If neutrophils succeed to surround pathogens (at low bacteria to PMNLs ratio), then after formation LTB_4_ is successfully transformed into ω-OH-LTB_4_. **(D)** When neutrophils meet pathogen clusters (high bacteria to PMNs ratio), LTB_4_ produced practically is not transformed to ω-OH-LTB_4_, and accumulated LTB_4_ signals for further neutrophil swarming.

We have demonstrated that synthesis of 5-LOX products induced by fMLP depends on bacteria:neutrophil ratio. With increase of bacteria load the ω-OH-LTB_4_/LTB_4_ ratio drops significantly (Figure 1E) indicating that bacteria not only increase the synthesis of LTB_4_, but also suppresses its transformation. This regulation can explain the behavior of the cells at neutrophil swarming (Scheme 1).

On bacterial infection, neutrophils leave the bloodstream and migrate to infection sites to eliminate bacterial pathogens. Neutrophils can engulf unopsonized microbes (Colucci-Guyon et al., 2011). Neutrophil swarming has been observed in several inflammatory and infectious conditions, ranging from sterile inflammation to infections. Pioneer neutrophils close to the damage site release signals to attract a second wave of neutrophils. Central to neutrophil swarming is a positive feedback amplification mechanism that is mediated by the LTB_4_ (Glaser et al., 2021). Neutrophil swarms manifest clearly under conditions of local injection of living bacteria into zebrafish larvae (Deng et al., 2013). As early released attractant the most interesting candidates are N-formyl peptides that can be released from damaged mitochondria of necrotic cells and are prominent inducers of chemotaxis.

In addition to well-known effects of bacteria and formyl peptides on neutrophils, this research identifies new ways of LTB_4_ synthesis regulation, and anti-inflammatory therapies targeting these inflammatory pathways must be tailored specifically based on the tissue LTB_4_/ω-OH-LTB_4_ profile.

## Data Availability

The original contributions presented in the study are included in the article/Supplementary Material, further inquiries can be directed to the corresponding author.

## References

[B1] Afonso P. V. Janka-Junttila M. Lee Y. J. McCann C. P. Oliver C. M. Aamer K. A. (2012). LTB4 Is a Signal-Relay Molecule during Neutrophil Chemotaxis. Dev. Cel 22, 1079–1091. 10.1016/j.devcel.2012.02.003 PMC4141281 22542839

[B2] Aleksandrov D. A. Zagryagskaya A. N. Pushkareva M. A. Bachschmid M. Peters-Golden M. Werz O. (2006). Cholesterol and its Anionic Derivatives Inhibit 5-lipoxygenase Activation in Polymorphonuclear Leukocytes and MonoMac6 Cells. FEBS J. 273, 548–557. 10.1111/j.1742-4658.2005.05087.x 16420478

[B3] Archambault A. S. Poirier S. Lefebvre J. S. Robichaud P. P. Larose M. C. Turcotte C. (2019). 20-Hydroxy- and 20-Carboxy-Leukotriene (LT) B4 Downregulate LTB4 -mediated Responses of Human Neutrophils and Eosinophils. J. Leukoc. Biol. 105, 1131–1142. 10.1002/JLB.MA0718-306R 30676680

[B4] Atlas S. J. Magargal W. W. Lin S. (1980). The Relationship between High-Affinity Binding of Cytochalasin B to 3T3 Cells and Inhibition of Sugar Transport and Cell Motility. J. Recept Res. 1, 113–135. 10.3109/10799898009044095 7299734

[B5] Bair A. M. Turman M. V. Vaine C. A. Panettieri R. A. Jr. Soberman R. J. (2012). The Nuclear Membrane Leukotriene Synthetic Complex Is a Signal Integrator and Transducer. Mol. Biol. Cel 23, 4456–4464. 10.1091/mbc.E12-06-0489 PMC3496618 23015755

[B6] Bauldry S. A. McCall C. E. Cousart S. L. Bass D. A. (1991). Tumor Necrosis Factor-Alpha Priming of Phospholipase A2 Activation in Human Neutrophils. An Alternative Mechanism of Priming. J. Immunol. 146, 1277–1285. 1846897

[B7] Beckman J. K. Gay J. C. Brash A. R. Lukens J. N. Oates J. A. (1985). Differential Effects of Lipoxygenase Products on FMLP and LTB4 Evoked Neutrophil Aggregation. Lipids 20, 357–360. 10.1007/BF02534202 2991693

[B8] Bloch R. (1973). Inhibition of Glucose Transport in the Human Erythrocyte by Cytochalasin B. Biochemistry 12, 4799–4801. 10.1021/bi00747a036 4773858

[B9] Brandt S. L. Serezani C. H. (2017). Too Much of a Good Thing: How Modulating LTB4 Actions Restore Host Defense in Homeostasis or Disease. Semin. Immunol. 33, 37–43. 10.1016/j.smim.2017.08.006 29042027 PMC5679129

[B10] Brideau C. Van Staden C. Styhler A. Rodger I. W. Chan C. C. (1999). The Effects of Phosphodiesterase Type 4 Inhibitors on Tumour Necrosis Factor-Alpha and Leukotriene B4 in a Novel Human Whole Blood Assay. Br. J. Pharmacol. 126, 979–988. 10.1038/sj.bjp.0702387 10193778 PMC1571215

[B11] Brock T. G. Paine R. 3rd Peters-Golden M. (1994). Localization of 5-lipoxygenase to the Nucleus of Unstimulated Rat Basophilic Leukemia Cells. J. Biol. Chem. 269, 22059–22066. 10.1016/s0021-9258(17)31755-6 8071328

[B12] Caffrey-Carr A. K. Hilmer K. M. Kowalski C. H. Shepardson K. M. Temple R. M. Cramer R. A. (2017). Host-Derived Leukotriene B4 Is Critical for Resistance against Invasive Pulmonary Aspergillosis. Front. Immunol. 8, 1984. 10.3389/fimmu.2017.01984 29375586 PMC5768911

[B13] Colucci-Guyon E. Tinevez J. Y. Renshaw S. A. Herbomel P. (2011). Strategies of Professional Phagocytes *In Vivo*: unlike Macrophages, Neutrophils Engulf Only Surface-Associated Microbes. J. Cel Sci 124, 3053–3059. 10.1242/jcs.082792 21868367

[B14] Dahlgren C. Gabl M. Holdfeldt A. Winther M. Forsman H. (2016). Basic Characteristics of the Neutrophil Receptors that Recognize Formylated Peptides, a Danger-Associated Molecular Pattern Generated by Bacteria and Mitochondria. Biochem. Pharmacol. 114, 22–39. 10.1016/j.bcp.2016.04.014 27131862

[B15] de Oliveira S. Rosowski E. E. Huttenlocher A. (2016). Neutrophil Migration in Infection and Wound Repair: Going Forward in Reverse. Nat. Rev. Immunol. 16, 378–391. 10.1038/nri.2016.49 27231052 PMC5367630

[B16] Deng Q. Sarris M. Bennin D. A. Green J. M. Herbomel P. Huttenlocher A. (2013). Localized Bacterial Infection Induces Systemic Activation of Neutrophils through Cxcr2 Signaling in Zebrafish. J. Leukoc. Biol. 93, 761–769. 10.1189/jlb.1012534 23475575 PMC4050646

[B17] DiPersio J. F. Billing P. Williams R. Gasson J. C. (1988). Human Granulocyte-Macrophage colony-stimulating Factor and Other Cytokines Prime Human Neutrophils for Enhanced Arachidonic Acid Release and Leukotriene B4 Synthesis. J. Immunol. 140, 4315–4322. 2453577

[B18] Doerfler M. E. Danner R. L. Shelhamer J. H. Parrillo J. E. (1989). Bacterial Lipopolysaccharides Prime Human Neutrophils for Enhanced Production of Leukotriene B4. J. Clin. Invest. 83, 970–977. 10.1172/JCI113983 2537852 PMC303773

[B19] Dorward D. A. Lucas C. D. Chapman G. B. Haslett C. Dhaliwal K. Rossi A. G. (2015). The Role of Formylated Peptides and Formyl Peptide Receptor 1 in Governing Neutrophil Function during Acute Inflammation. Am. J. Pathol. 185, 1172–1184. 10.1016/j.ajpath.2015.01.020 25791526 PMC4419282

[B20] Fischer J. Gresnigt M. S. Werz O. Hube B. Garscha U. (2021). Candida Albicans-Induced Leukotriene Biosynthesis in Neutrophils Is Restricted to the Hyphal Morphology. FASEB J. 35, e21820. 10.1096/fj.202100516RR 34569657 PMC12316086

[B21] Földes-Filep E. Filep J. G. (1992). Mepacrine Inhibits fMLP-Induced Activation of Human Neutrophil Granulocytes, Leukotriene B4 Formation, and fMLP Binding. J. Leukoc. Biol. 52, 545–550. 10.1002/jlb.52.5.545 1331280

[B22] Galkina S. I. Fedorova N. V. Serebryakova M. V. Arifulin E. A. Stadnichuk V. I. Gaponova T. V. (2015). Inhibition of the GTPase Dynamin or Actin Depolymerisation Initiates Outward Plasma Membrane Tubulation/vesiculation (Cytoneme Formation) in Neutrophils. Biol. Cel 107, 144–158. 10.1111/boc.201400063 25655190

[B23] Gerstmeier J. Weinigel C. Rummler S. Rådmark O. Werz O. Garscha U. (2016). Time-resolved *In Situ* Assembly of the Leukotriene-Synthetic 5-Lipoxygenase/5-Lipoxygenase-Activating Protein Complex in Blood Leukocytes. FASEB J. 30, 276–285. 10.1096/fj.15-278010 26396238

[B24] Glaser K. M. Mihlan M. Lämmermann T. (2021). Positive Feedback Amplification in Swarming Immune Cell Populations. Curr. Opin. Cel Biol 72, 156–162. 10.1016/j.ceb.2021.07.009 34500367

[B25] Golenkina E. A. Galkina S. I. Romanova J. M. Lazarenko M. I. Sud'ina G. F. (2011). Involvement of Red Blood Cells in the Regulation of Leukotriene Synthesis in Polymorphonuclear Leucocytes upon Interaction with Salmonella Typhimurium. APMIS 119, 635–642. 10.1111/j.1600-0463.2011.02786.x 21851422

[B26] Graham F. D. Erlemann K. R. Gravel S. Rokach J. Powell W. S. (2009). Oxidative Stress-Induced Changes in Pyridine Nucleotides and Chemoattractant 5-lipoxygenase Products in Aging Neutrophils. Free Radic. Biol. Med. 47, 62–71. 10.1016/j.freeradbiomed.2009.04.016 19376220 PMC2891157

[B27] Gröne M. Scheffer J. König W. (1992). Modulation of Leukotriene Generation by Invasive Bacteria. Immunology 77, 400–407. 1335960 PMC1421725

[B28] Haeggström J. Z. (2018). Leukotriene Biosynthetic Enzymes as Therapeutic Targets. J. Clin. Invest. 128, 2680–2690. 10.1172/JCI97945 30108195 PMC6026001

[B29] Harbecke O. Liu L. Karlsson A. Dahlgren C. (1997). Desensitization of the fMLP-Induced NADPH-Oxidase Response in Human Neutrophils Is Lacking in Okadaic Acid-Treated Cells. J. Leukoc. Biol. 61, 753–758. 10.1002/jlb.61.6.753 9201267

[B30] Hind L. E. Giese M. A. Schoen T. J. Beebe D. J. Keller N. Huttenlocher A. (2021). Immune Cell Paracrine Signaling Drives the Neutrophil Response to A. fumigatus in an Infection-On-A-Chip Model. Cell Mol Bioeng 14, 133–145. 10.1007/s12195-020-00655-8 33868496 PMC8010091

[B31] Hopke A. Scherer A. Kreuzburg S. Abers M. S. Zerbe C. S. Dinauer M. C. (2020). Neutrophil Swarming Delays the Growth of Clusters of Pathogenic Fungi. Nat. Commun. 11, 2031. 10.1038/s41467-020-15834-4 32341348 PMC7184738

[B32] Jesaitis A. J. Erickson R. W. Klotz K. N. Bommakanti R. K. Siemsen D. W. (1993). Functional Molecular Complexes of Human N-Formyl Chemoattractant Receptors and Actin. J. Immunol. 151, 5653–5665. 8228254

[B33] Jesaitis A. J. Klotz K. N. (1993). Cytoskeletal Regulation of Chemotactic Receptors: Molecular Complexation of N-Formyl Peptide Receptors with G Proteins and Actin. Eur. J. Haematol. 51, 288–293. 10.1111/j.1600-0609.1993.tb01610.x 8282090

[B34] Karlsson A. Markfjäll M. Strömberg N. Dahlgren C. (1995). Escherichia Coli-Induced Activation of Neutrophil NADPH-Oxidase: Lipopolysaccharide and Formylated Peptides Act Synergistically to Induce Release of Reactive Oxygen Metabolites. Infect. Immun. 63, 4606–4612. 10.1128/IAI.63.12.4606-4612.1995 7591113 PMC173662

[B35] Kienle K. Glaser K. M. Eickhoff S. Mihlan M. Knöpper K. Reátegui E. (2021). Neutrophils Self-Limit Swarming to Contain Bacterial Growth *In Vivo* . Science 372. 10.1126/science.abe7729 PMC8926156 34140358

[B36] Klotz K. N. Jesaitis A. J. (1994). Physical Coupling of N-Formyl Peptide Chemoattractant Receptors to G Protein Is Unaffected by Desensitization. Biochem. Pharmacol. 48, 1297–1300. 10.1016/0006-2952(94)90168-6 7945424

[B37] Klotz K. N. Krotec K. L. Gripentrog J. Jesaitis A. J. (1994). Regulatory Interaction of N-Formyl Peptide Chemoattractant Receptors with the Membrane Skeleton in Human Neutrophils. J. Immunol. 152, 801–810. 8283053

[B38] Kobayashi S. D. Malachowa N. DeLeo F. R. (2018). Neutrophils and Bacterial Immune Evasion. J. Innate Immun. 10, 432–441. 10.1159/000487756 29642066 PMC6784029

[B39] Kulkarni S. Das S. Funk C. D. Murray D. Cho W. (2002). Molecular Basis of the Specific Subcellular Localization of the C2-like Domain of 5-lipoxygenase. J. Biol. Chem. 277, 13167–13174. 10.1074/jbc.M112393200 11796736

[B40] Lämmermann T. Afonso P. V. Angermann B. R. Wang J. M. Kastenmüller W. Parent C. A. (2013). Neutrophil Swarms Require LTB4 and Integrins at Sites of Cell Death *In Vivo* . Nature 498, 371–375. 10.1038/nature12175 23708969 PMC3879961

[B41] Luo M. Jones S. M. Peters-Golden M. Brock T. G. (2003). Nuclear Localization of 5-lipoxygenase as a Determinant of Leukotriene B4 Synthetic Capacity. Proc. Natl. Acad. Sci. U S A. 100, 12165–12170. 10.1073/pnas.2133253100 14530386 PMC218730

[B42] Mancuso P. Nana-Sinkam P. Peters-Golden M. (2001). Leukotriene B4 Augments Neutrophil Phagocytosis of *Klebsiella pneumoniae* . Infect. Immun. 69, 2011–2016. 10.1128/IAI.69.4.2011-2016.2001 11254552 PMC98124

[B43] Mandal A. K. Jones P. B. Bair A. M. Christmas P. Miller D. Yamin T. T. (2008). The Nuclear Membrane Organization of Leukotriene Synthesis. Proc. Natl. Acad. Sci. U S A. 105, 20434–20439. 10.1073/pnas.0808211106 19075240 PMC2629249

[B44] Metschnikoff E. (1891). Lecture on Phagocytosis and Immunity. Br. Med. J. 1, 213–217. 10.1136/bmj.1.1570.213 PMC2197023 20753232

[B45] Miralda I. Uriarte S. M. McLeish K. R. (2017). Multiple Phenotypic Changes Define Neutrophil Priming. Front Cel Infect Microbiol 7, 217. 10.3389/fcimb.2017.00217 PMC5447094 28611952

[B46] Nadeau M. Fruteau de Laclos B. Picard S. Braquet P. Corey E. J. Borgeat P. (1984). Studies on Leukotriene B4 omega-oxidation in Human Leukocytes. Can J. Biochem. Cel Biol 62, 1321–1326. 10.1139/o84-168 6099214

[B47] Newcomer M. E. Gilbert N. C. (2010). Location, Location, Location: Compartmentalization of Early Events in Leukotriene Biosynthesis. J. Biol. Chem. 285, 25109–25114. 10.1074/jbc.R110.125880 20507998 PMC2919072

[B48] Nielsen H. Andersen L. P. (1992). Activation of Human Phagocyte Oxidative Metabolism by *Helicobacter pylori* . Gastroenterology 103, 1747–1753. 10.1016/0016-5085(92)91430-c 1451968

[B49] Palmantier R. Surette M. E. Sanchez A. Braquet P. Borgeat P. (1994). Priming for the Synthesis of 5-lipoxygenase Products in Human Blood *Ex Vivo* by Human Granulocyte-Macrophage colony-stimulating Factor and Tumor Necrosis Factor-Alpha. Lab. Invest. 70, 696–704. 8196365

[B50] Plagge M. Laskay T. (20172017). Early Production of the Neutrophil-Derived Lipid Mediators LTB4 and LXA4 Is Modulated by Intracellular Infection with Leishmania Major. Biomed. Res. Int. 2017, 2014583. 10.1155/2017/2014583 PMC5664244 29181388

[B51] Poplimont H. Georgantzoglou A. Boulch M. Walker H. A. Coombs C. Papaleonidopoulou F. (2020). Neutrophil Swarming in Damaged Tissue Is Orchestrated by Connexins and Cooperative Calcium Alarm Signals. Curr. Biol. 30, 2761–e7. 10.1016/j.cub.2020.05.030 32502410 PMC7372224

[B52] Powell W. S. (1984). Properties of Leukotriene B4 20-hydroxylase from Polymorphonuclear Leukocytes. J. Biol. Chem. 259, 3082–3089. 10.1016/s0021-9258(17)43263-7 6321494

[B53] Rådmark O. Samuelsson B. (2010). Regulation of the Activity of 5-lipoxygenase, a Key Enzyme in Leukotriene Biosynthesis. Biochem. Biophys. Res. Commun. 396, 105–110. 10.1016/j.bbrc.2010.02.173 20494120

[B54] Rådmark O. Werz O. Steinhilber D. Samuelsson B. (2015). 5-Lipoxygenase, a Key Enzyme for Leukotriene Biosynthesis in Health and Disease. Biochim. Biophys. Acta 1851, 331–339. 10.1016/j.bbalip.2014.08.012 25152163

[B55] Reátegui E. Jalali F. Khankhel A. H. Wong E. Cho H. Lee J. (2017). Microscale Arrays for the Profiling of Start and Stop Signals Coordinating Human-Neutrophil Swarming. Nat. Biomed. Eng. 1. 10.1038/s41551-017-0094 PMC5646699 29057147

[B56] Rocha-Gregg B. L. Huttenlocher A. (2021). Swarming Motility in Host Defense. Science 372, 1262–1263. 10.1126/science.abj3065 34140369

[B57] Romp E. Arakandy V. Fischer J. Wolz C. Siegmund A. Löffler B. (2020). Exotoxins from *Staphylococcus aureus* Activate 5-lipoxygenase and Induce Leukotriene Biosynthesis. Cell Mol Life Sci 77, 3841–3858. 10.1007/s00018-019-03393-x 31807813 PMC11105070

[B58] Schiffmann E. Corcoran B. A. Wahl S. M. (1975). N-formylmethionyl Peptides as Chemoattractants for Leucocytes. Proc. Natl. Acad. Sci. U S A. 72, 1059–1062. 10.1073/pnas.72.3.1059 1093163 PMC432465

[B59] Snyderman R. Pike M. C. (1984). Chemoattractant Receptors on Phagocytic Cells. Annu. Rev. Immunol. 2, 257–281. 10.1146/annurev.iy.02.040184.001353 6100474

[B60] Surette M. E. Palmantier R. Gosselin J. Borgeat P. (1993). Lipopolysaccharides Prime Whole Human Blood and Isolated Neutrophils for the Increased Synthesis of 5-lipoxygenase Products by Enhancing Arachidonic Acid Availability: Involvement of the CD14 Antigen. J. Exp. Med. 178, 1347–1355. 10.1084/jem.178.4.1347 7690833 PMC2191210

[B61] Vénéreau E. Ceriotti C. Bianchi M. E. (2015). DAMPs from Cell Death to New Life. Front. Immunol. 6, 422. 10.3389/fimmu.2015.00422 26347745 PMC4539554

[B62] Viryasova G. M. Galkina S. I. Gaponova T. V. Romanova J. M. Sud'ina G. F. (2014). Regulation of 5-Oxo-ETE Synthesis by Nitric Oxide in Human Polymorphonuclear Leucocytes upon Their Interaction with Zymosan and *Salmonella typhimurium* . Biosci. Rep. 34. 10.1042/BSR20130136 PMC4031671 24712762

[B63] Viryasova G. M. Golenkina E. A. Galkina S. I. Gaponova T. V. Romanova Y. M. Sud'ina G. F. (2016). Effects of Phosphodiester and Phosphorothioate ODN2216 on Leukotriene Synthesis in Human Neutrophils and Neutrophil Apoptosis. Biochimie 125, 140–149. 10.1016/j.biochi.2016.03.010 27036535

[B64] Werz O. Gerstmeier J. Libreros S. De la Rosa X. Werner M. Norris P. C. (2018). Human Macrophages Differentially Produce Specific Resolvin or Leukotriene Signals that Depend on Bacterial Pathogenicity. Nat. Commun. 9, 59. 10.1038/s41467-017-02538-5 29302056 PMC5754355

[B65] Yamamoto S. Adjei A. A. Kise M. (1993). Intraperitoneal Administration of Leukotriene B4 (LTB4) and omega-guanidino Caproic Acid Methane Sulfonate (GCA) Increased the Survival of Mice Challenged with Methicillin-Resistant *Staphylococcus aureus* (MRSA). Prostaglandins 45, 527–534. 10.1016/0090-6980(93)90016-z 8393205

[B66] Ye R. D. Boulay F. Wang J. M. Dahlgren C. Gerard C. Parmentier M. (2009). International Union of Basic and Clinical Pharmacology. LXXIII. Nomenclature for the Formyl Peptide Receptor (FPR) Family. Pharmacol. Rev. 61, 119–161. 10.1124/pr.109.001578 19498085 PMC2745437

[B67] Yuli I. Snyderman R. (1986). Extensive Hydrolysis of N-Formyl-L-Methionyl-L-Leucyl-L-[3h] Phenylalanine by Human Polymorphonuclear Leukocytes. A Potential Mechanism for Modulation of the Chemoattractant Signal. J. Biol. Chem. 261, 4902–4908. 10.1016/s0021-9258(19)89190-1 3957916

