# Supplementary material for: Gram-Negative Bacteria Salmonella typhimurium Boost Leukotriene Synthesis Induced by Chemoattractant fMLP to Stimulate Neutrophil Swarming
Source: Front Pharmacol. 2022 Jan 4;12:814113. doi: 10.3389/fphar.2021.814113 (PMC8764451; doi:10.3389/fphar.2021.814113)
Supplement: Supplementary file 1 [file DataSheet1.pdf]

frontiers

Supplementary Material

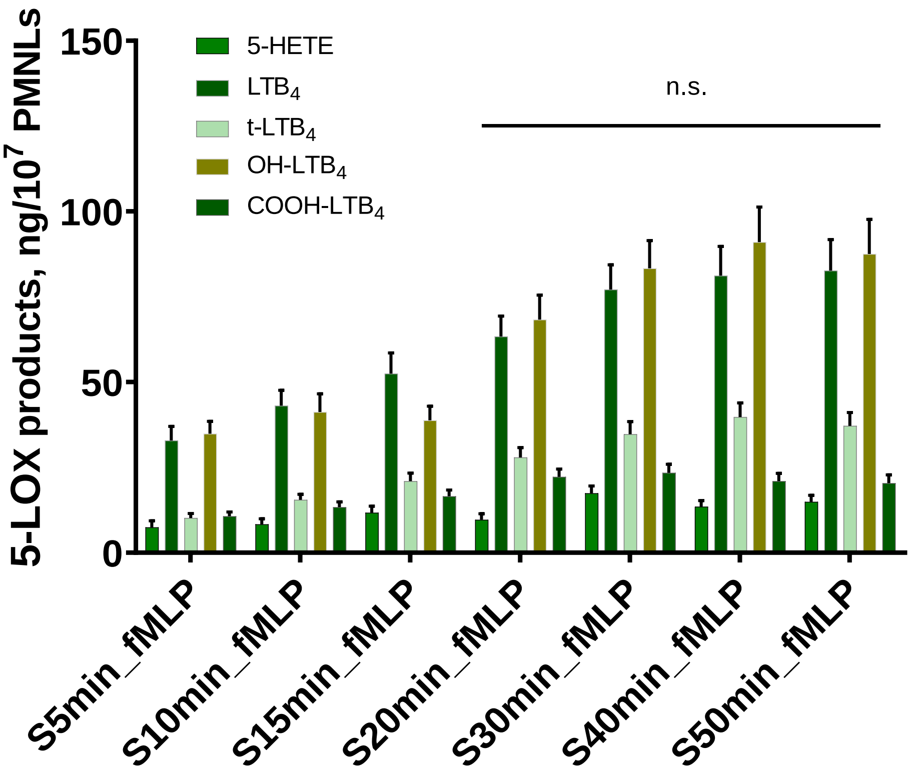

Supplementary Figure 1. 5-LOX product synthesis in human neutrophil at bacteria *Salmonella typhimurium* exposure (the ratio of bacteria:PMNLs ~ 25:1) followed by fMLP (0.1 µM) addition for 10 min. PMNLs (0.9-1.0) x10^7/6 ml were pre-incubated for 10 min at 37 ^{o}C, 5% CO_2. Bacteria were added for 5-50 min, the time is indicated. The 5-LOX products were analyzed using HPLC, and data for 5-HETE, LTB_4, t-LTB_4, ω-OH-LTB_4 and ω-COOH-LTB_4 are presented. Values indicate mean ± SEM of three independent experiments performed in duplicate. The pairs of data were compared by two-way ANOVA followed by Tukey's multiple comparison test.

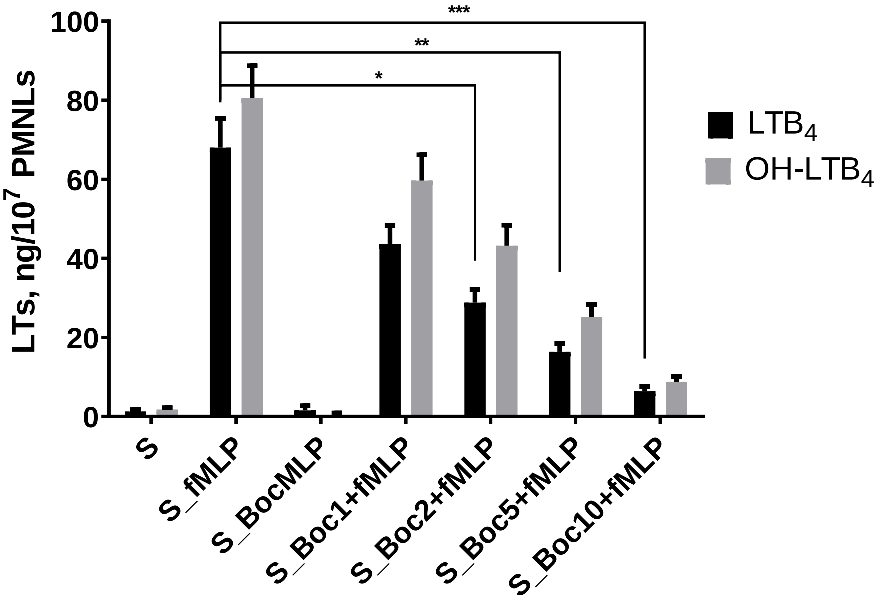

Supplementary Figure 2. Effect of BocMLP (Boc) on leukotriene synthesis in human neutrophil at bacteria *Salmonella typhimurium* (S) exposure followed by fMLP (0.1 µM), or BocMLP (0.1 µM) or BocMLP and fMLP addition for 10 min. The ratio of bacteria:PMNLs ~ 25:1. PMNLs (0.9-1.0) x10^7/6ml were pre-incubated for 10 min at 37 °C, 5% CO_2. At single treatment, S were added for 30 min. At complex treatment, bacteria were added for 30 min, then fMLP (0.1 µM), or BocMLP (the number means 1, 2, 5, 10 µM) and immediately fMLP were added for 10 min. The 5-LOX products were analyzed using HPLC, and data for LTB_4 and ω-OH-LTB_4 are presented. Values indicate mean ± SEM of three independent experiments performed in duplicate. *p < 0.05, **p < 0.01, ***p < 0.001 for pairs of data compared as indicated by two-way ANOVA followed by Tukey’s multiple comparison test.

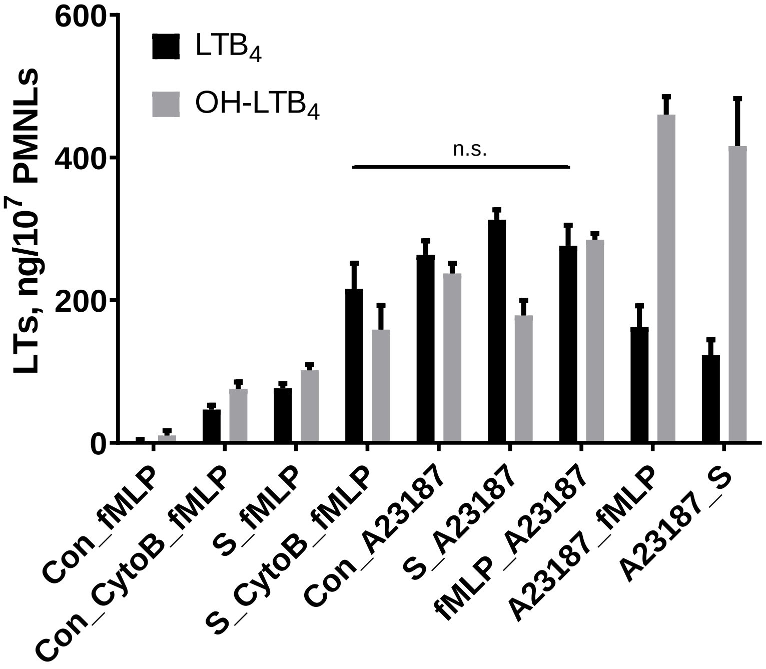

Supplementary Figure 3. Effect of various stimuli on leukotriene synthesis in human neutrophils. PMNLs (0.9-1.0) x10^7/6ml were pre-incubated for 10 min at 37 °C, 5% CO_2. First, PMNLs incubated with no additives (Con) or bacteria (S) or fMLP (0.1 µM) or A23187 (1 µM) for 30min; then Cyto B (5 µM) was added for 10 min, as indicated. Further, fMLP (0.1 µM) or A23187 (1 µM) or bacteria were added for 10 min, as indicated. The 5-LOX products were analyzed using HPLC, and data for LTB4 and ω-OH-LTB4 are presented. Values indicate mean ± SEM of four independent experiments performed in duplicate. The pairs of data compared by two-way ANOVA followed by Tukey's multiple comparison test.

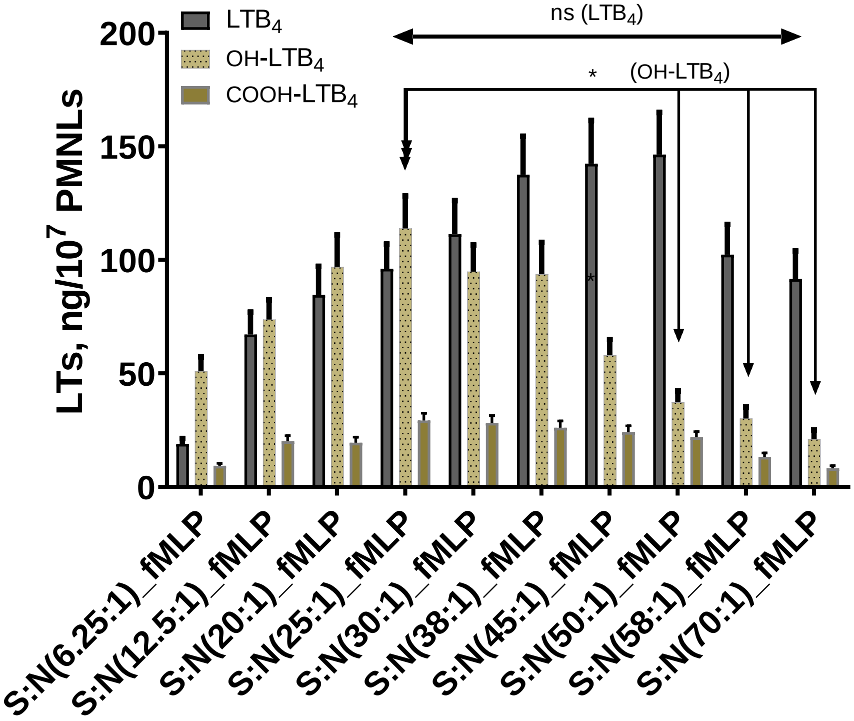

Supplementary Figure 4. Leukotriene synthesis in human neutrophils at various bacterial load. PMNLs (0.9-1.0) x10^7/6 ml were pre-incubated for 10 min at 37 ^{o}C, 5% CO_2. Human neutrophils exposed to *Salmonella typhimurium* for 30 min (1st treatment) followed by fMLP (0.1 µM) addition for 10 min, the ratio of bacteria (S):PMNLs (N) is indicated. The 5-LOX products were analyzed using HPLC, and data for LTB_4, ω-OH-LTB_4 and ω-COOH-LTB_4 are presented. Values indicate mean ± SEM of three independent experiments performed in duplicate. *$p < 0.05$ for pairs of data compared as indicated by two-way ANOVA followed by Tukey's multiple comparison test.

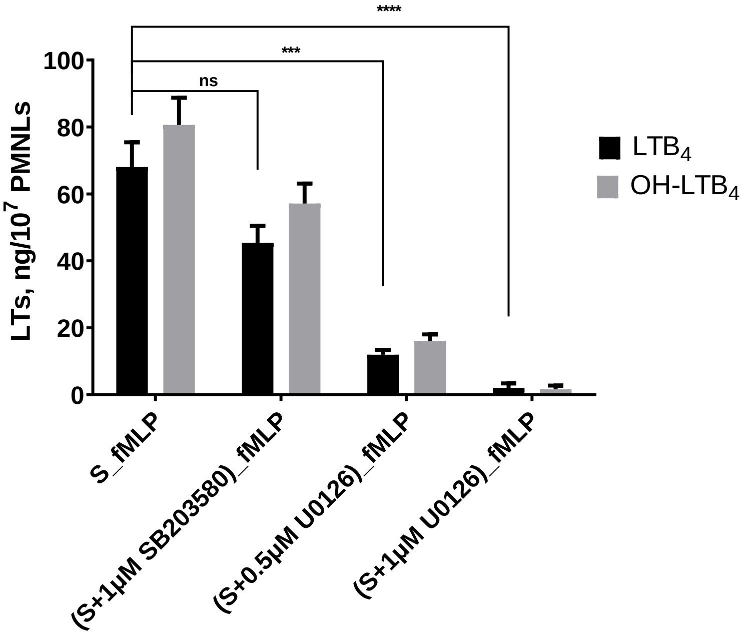

Supplementary Figure 5. Effect of MAPK inhibitors SB203580 and U0126 on leukotriene synthesis in human neutrophil at bacteria *Salmonella typhimurium* (S) exposure followed by fMLP (0.1 µM) addition. The ratio of bacteria:PMNLs ~ 25:1. PMNLs (0.9-1.0) x10^7/6 ml were pre-incubated for 10 min at 37 °C, 5% CO_2. At 1st treatment, bacteria, or bacteria plus SB203580, or bacteria plus U0126 were added for 30 min, then fMLP (0.1 µM) was added for 10 min. The 5-LOX products were analyzed using HPLC, and data for LTB_4 and ω-OH-LTB_4 are presented. Values indicate mean ± SEM of three independent experiments performed in duplicate. ***$p < 0.001$, ****$p < 0.0001$ for pairs of data compared as indicated by two-way ANOVA followed by Tukey's multiple comparison test.
